# 

**Published:** 1887-05

**Authors:** 


CONTENTS:

Article I.—A Factor in Tooth Preservation. By Prof. C. N. Pierce..... 1
" II.—Electricity and its Application to Dentistry. By Dr. George C. Brown..... 14
" III.—Alveolar Abscess..... 21
" IV.—Implantation..... 24
" V.—Extract from a Monograph on Local Anæsthesia in Dentistry and Minor Surgery. By. Prof. George Viau; translated by L. Turnbull, M. D .. 30
" VI —Metal Work. By Dr. L. P. Haskell..... 35
" VII.—The Matrix in Filling Teeth ..... 37

EDITORIAL, ETC.

Daily Life in a Dental Infirmary..... 39
The Value of a Name in Dentistry ..... 43

BIBLIOGRAPHICAL.

A System of Dental Surgery..... 44

MONTHLY SUMMARY.

A Method of Filling Very Fine Root Canals..... 45

A Method of Bridge-Work ..... 46

Bleaching Teeth..... 47

Teething..... 48

